# Protective Role of Glucagon-Like Peptide-1 Against High-Glucose-Induced Endothelial Oxidative Damage

**DOI:** 10.1097/MD.0000000000002055

**Published:** 2015-10-30

**Authors:** Lixin Guo, Yue Qiao, Lina Zhang, Qi Pan

**Affiliations:** From the Department of Endocrinology, Beijing Hospital, Beijing, P.R. China.

## Abstract

To investigate the protective effect of glucagon-like peptide-1 (GLP-1) against cell damage induced by high glucose.

Human umbilical vein endothelial cells (HUVECs) were divided into control group (5.5 mmol/L) and high glucose groups (19, 33, or 47 mmol/L), which were cultured with different concentrations of glucose for 48 hours, respectively. Cell viability was measured with MTT assay. Levels of intracellular reactive oxygen species (ROS) were monitored by flow cytometry and apoptotic cell death was measured by staining with Annexin V-FITC and propidium iodide. Cultured cells were detected with intercellular adhesion molecule 1 (ICAM-1), VCAM-1, and JNK on protein.

Compared with the control group, cell viability was decreased by 20% and 37%, respectively, when cultured under 33 and 47 mM, while increased in different GLP-1-treated groups (0.01 L, 0.1, 1, and 10 nmol/L). The GLP-1 treatment significantly reduced the ROS level of high glucose treatment group but not impact on the control group. Meanwhile, the level of apoptosis was elevated in the high glucose treatment group. Early apoptosis was significantly reversed in the GLP-1-treated group (0.1, 1, and 10 nmol/L). Late apoptosis was uniquely decreased in the GLP-1 concentrations of 10 nmol/L. Furthermore, GLP-1 could also reduce the protein levels of ICAM-1, VCAM-1, and phospho JNK in the endothelial cells with high glucose treatment.

GLP-1 could inhibit cell apoptosis and reduce ROS generation and JNK-Bax signaling pathway activation, which were induced by high glucose treatment.

## INTRODUCTION

Hyperglycemia is the symbol of diabetes and also linked to macrovascular complications.^1^ Vascular endothelial cells play important roles in maintaining the vascular function, while endothelial dysfunction contributes to the pathogenesis of vascular diseases in diabetes.^2^ As we known, hyperglycemia is associated with endothelial cell dysfunction in diabetes and might be one of the causes of premature atherosclerosis.^2^ It was suggested that oxidative stress and production of reactive oxygen species (ROS) induced by chronic hyperglycemia play a key role in diabetic progression.^3–5^ The role of ROS in the pathogenesis of diabetes mellitus is quite recognized as modification of various cellular events in many tissues and cells including vessels, kidney, pancreatic beta cells, and liver. The ROS increases intracellular (DNA) damage and ultimately results in the onset of apoptosis or the induction of cell senescence.^6^ Thus, inhibition of ROS generation may represent an effective strategy to reverse the cell injury. However, the molecular basis of this signaling pathway is still unclear. Intercellular adhesion molecule 1 (ICAM-1)/vascular cell adhesion molecule 1 (VCAM-1) is a transmembrane glycoprotein, which is a member of the immunoglobulin gene super family. These molecules play important roles in the adhesion of circulating leucocytes to the endothelium, which is the first step of atherosclerosis initiation.^7^ C-Jun N-terminal kinase (JNK) family is a member of mitogen-activated protein kinase (MAPK) superfamily. The JNK signal pathway can be activated by cytokines, growth factors, stress, and so on. JNK activity can regulate several important cellular functions including cell growth, differentiation, survival, and apoptosis. Apoptosis regulator Bax is a member of the Bcl-2 gene family. This protein plays an important role in the activation of apoptosis and can be regulated by the tumor suppressor P53, which is involved in P53-mediated apoptosis.

Glucagon-like peptide-1 (GLP-1) is an incretin that derived from the transcription product of the proglucagon gene, and is secreted mainly from intestinal L cells in response to the presence of nutrients as a gut hormone, which can stimulate the glucose-dependent insulin secretion in β-cell^8^ and activate anti-apoptotic signaling pathways in pancreatic cells. GLP-1 receptor is a member of the Gs-protein-coupled receptor superfamily, which is detected in the gastrointestinal tract nervous system, heart, vascular smooth muscle, adipose tissues, and endothelial cells.^9–11^ Previous studies revealed that GLP-1 could protect against vascular endothelial cells injured by high glucose^12–14^ and decrease the ROS production.^14,15^ Also, some studies showed that GLP-1 could inhibit high-glucose induced oxidative stress and cell apoptosis in HUVECs through GLP-1R-dependent and GLP-1 related pathways.^16^ GLP-1 has been proposed to be a potential therapeutic target for the treatment of patients with type II diabetes. However, the direct effect mechanism of GLP-1 on vascular injury in diabetes and its relationship with ROS and downstream signaling pathway is largely unknown.^17^ To determine the role of GLP-1 in oxidative stress and apoptosis induced by high glucose, we paired cultures of human umbilical vein endothelial cells (HUVECs) isolates that were exposed to high concentration of glucose and GLP-1 in order to investigate the effects of GLP-1 on oxidative stress and cell apoptosis induced by high concentration of glucose.

## MATERIALS AND METHODS

### Materials

The M199 and fetal bovine serum were from Hyclone (Logan, UT). d-glucose, GLP-1(7-36), MTT, and collagenase I were purchased from Sigma Aldrich (Shanghai, China). Endothelial cell growth factor (ECGF) was purchased from Roche (Shanghai, China). β-actin polyclonal antibody, rabbit anti-goat JNK, p-JNK, Bax, and polyclonal antibody were purchased from Cell Signal Technology (Danvers, MA). The rabbit anti-goat ICAM-1 and VCAM-1 polyclonal antibodies were purchased from Santa Cruz Biotechnology (Santa Cruz, CA). Horseradish peroxidase (HRP) labeled goat anti-rabbit secondary antibody was from Zhongshan (Beijing, China). Annexin V-FITC and PI apoptosis detection kit was purchased from Baosai (Beijing, China). Western Blotting Luminol Reagent and poly(vinylidene fluoride) (PVDF) membranes were purchased from Millipore (Billerica, MA).

### Tissue Preparation and Cell Cultures

This study was approved by the Medical Ethics Committee of Beijing Hospital. HUVECs were isolated from newborn umbilical veins with collagenase treatment and were cultured on gelatin-coated culture dishes in Medium 199 containing 20% fetal bovine serum supplemented with penicillin/streptomycin, at 37°C in humidified 5% CO_2_ in air. Cells from the passages 3 to 5 were mainly used in this study. Human tissues were treated according to the Declaration of Helsinki. Written informed consent was obtained from all participant patients.

### Detection of Intracellular ROS Production

The ROS production was monitored by flow cytometry (FACSCalibur Becton-Dickinson, Franklin Lakes, NJ) using 2,7-dichlorofluorescin diacetate (DCFH-DA, Sigma Aldrich). Briefly, the cells were treated with GLP-1 in different glucose concentrations of medium for predetermined periods and then coincubated with 10 μmol/L DCFH-DA. After incubation, the cells were resuspended in ice-cold PBS and were kept in the dark for flow cytometry analysis.

### Determination of Apoptosis

Apoptosis of cells were quantitatively detected using Annexin V-FITC and PI apoptosis detection kit (Bao Sai, Beijing China). Briefly, the cells were seeded into 6-well plates (5.0 × 10^5^ cells/mL) and incubated for 24 hours. The cells were treated with the different concentrations of glucose and GLP-1 for varying times, harvested and washed twice with ice-cold PBS. After 5 minutes of centrifuging at 5000 rpm, Annexin V-FITC and PI double-staining were performed according to the manufacturer's instructions. Annexin V-FITC-positive but PI-negative cells were scored as early apoptotic ones. Double-stained cells were considered as late apoptotic and necrotic ones. The percentage of normal, early apoptotic, late apoptotic, and necrotic cells were calculated using FACS Calibur and Cell Quest software (Becton-Dickinson), respectively.^18^

### MTT Assay

Cell viability was measured with 3-(4,5-dimethyl-thiazol-2-yl)-2,5-diphenyl-tetrazolium bromide (MTT) assay, which were known as a colorimetric assay for assessing cell metabolic activity. Briefly, HUVECs were seeded in 96-well plates (1.0 × 10^5^ cells/mL) and incubated in M199 medium for 24 hours. They were then incubated with the glucose and GLP-1 for the indicated time. The MTT reagent (5 mg/mL) was added to each of the wells, and the plate was incubated for an additional 4 hours at 37°C. The media were removed and the intracellular formazan product was dissolved in 150 μL of DMSO. The absorption value of each well was then measured at 540 nm using the microplate reader (Spectra Max 190, Molecular Devices, Sunnyvale, California, USA).

### Western Blot Analysis

The protein levels of JNK, VCAM-1, ICAM-1, Bax, and the phospho JNK (p-JNK) were analyzed by Western blotting. The lysis buffer was prepared and included: HC (l50 mmol/L, pH 7.6), NaCl (150 mmol/L), 1% NP-40, 0.1% sodium dodecyl sulfate (SDS), dithiothreitol (1 mmol/L), sodium vanadate (1 mmol/L), phenylmethylsulfonyl fluoride (1 mmol/L), aprotinin (10 μg/mL), leupeptin (10 μg/mL), and sodium fluoride (10 mmol/L). The cells were placed in the lysis buffer on ice for 30 min. Equal amounts of protein were separated by SDS–polyacrylamide gel electrophoresis and were transferred to nitrocellulose filters. The membrane was blocked in buffer containing BSA (1%) and Tween 20 (0.1%, v/v) in PBS (PBS/Tween 20) at room temperature for 1 h. It was incubated overnight at 4°C with anti-JNK, anti-p-JNK, anti-VCAM-1, and ICAM-1 polyclonal antibody. Next, they were incubated with the proper secondary antibodies at room temperature for 2 hours. Finally, each membrane was developed using an enhanced ChemiImager 5500 chemiluminescence system (Alpha Innotech Corporation, Miami, FL).

### Statistical Analysis

Data were presented as mean ± SD. All statistical data were obtained by one-way ANOVA followed by Student *t* test. Statistical significance was assigned at the level of *P* < 0.05.

## RESULTS

### GLP-1 Increased Cell Viability in HUVECs Treated With High Glucose

**FIGURE 1:**
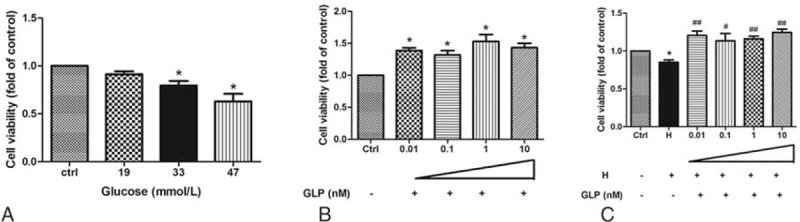
The change of cell activity treated with different concentrations of glucose and GLP-1 in HUVECs. (A) HUVECs were treated with different concentrations of glucose. (B) Control groups treated with GLP-1. (C) HUVECs were treated with high glucose and GLP-1 and were compared with the control group (^∗,#^*P* < 0.05, ^∗∗,##^*P* < 0.01). Note: Ctrl: control (5.5 mmol/L glucose); H: 33 mmol/L glucose; ^∗^: compare with control group; #: compare with H group.

We investigated the cell viability in different concentrations of high glucose group. Compared with the control group, cell viability was decreased in the group treated with different concentrations of glucose (Fig. 1A). We further investigated whether GLP-1 could actually change cell viability in the HUVECs. Compared with the control group, GLP-1 could increase the cell viability in a dose-dependent manner with different GLP-1 concentrations (Fig. 1B). Correspondingly, GLP could also reverse cell viability inhibited by high glucose (Fig. 1C).

### GLP-1 Inhibited the ROS Generation Induced by High Glucose in HUVECs

**FIGURE 2:**
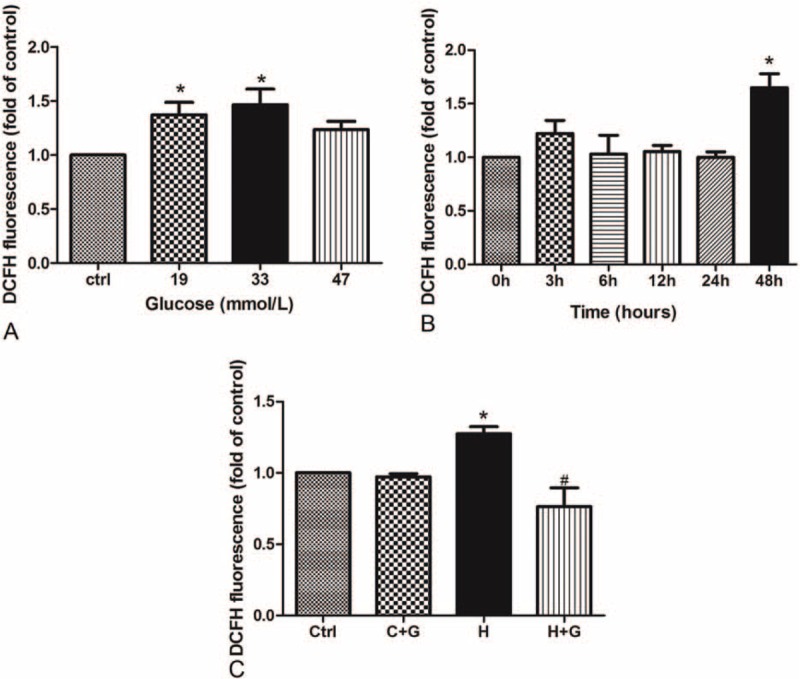
GLP-1 inhibited the ROS production induced by high concentration glucose in HUVECs. (A) High concentration of glucose increased the ROS generation in HUVECs treated in 48 hours; (B) HUVECs were treated with 33 mmol/L glucose in different times; (C) GLP-1 decreased the ROS induced by high glucose in HUVECs as compared with control group (^∗^*P* < 0.05, compare with C group; ^#^*P* < 0.05, compare with H group). Note: Ctrl: control (5.5 mmol/L glucose); C + G: control + 10 nmol/L GLP-1; H: 33 mmol/L glucose; H + G: 33 mmol/L glucose + 10 nmol/L GLP-1.

HUVECs were treated with high concentrations of glucose and the increased levels of ROS were measured by DCFH fluorescence in a time- and dose-dependent manner. Compared with the control group, the treatment groups with 19 and 33 mmol/L glucose had a significant induction of ROS (Fig. 2A). The levels of ROS were detected in the cells treated with 33 mmol/L glucose in different time points (0, 3, 6, 12, 24, and 48 hours). Compared with the 0 hour group, the group treated with glucose in 48 hours had an increased level of ROS (Fig. 2B). We next investigated whether GLP-1 could actually decrease the ROS production in HUVECs. The results showed that GLP-1 could significantly reduce the ROS level of the high glucose group (Fig. 2C).

**FIGURE 3:**
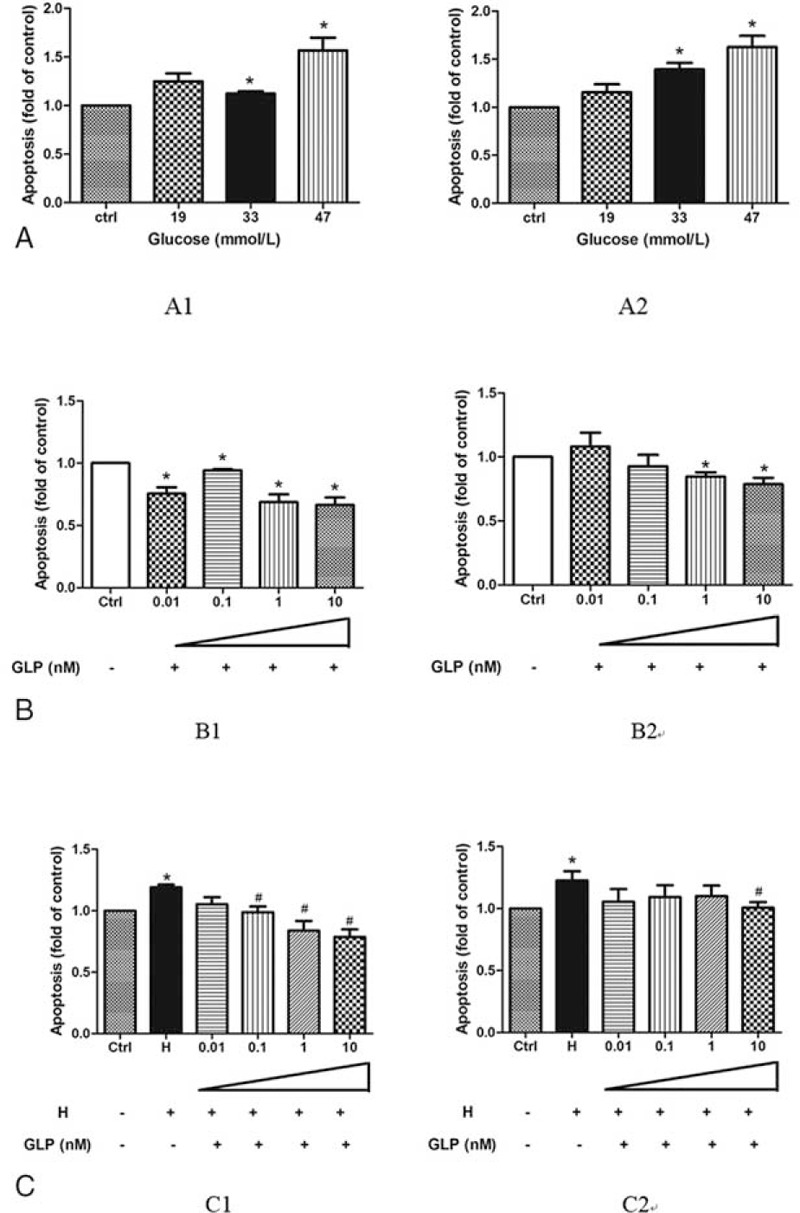
GLP-1 inhibited the high glucose inducible apoptosis in HUVECs. (A) HUVECs were treated with different concentrations of glucose (A1: early apoptosis, A2: late apoptosis); (B) HUVECs were treated with different concentration of GLP-1 (B1: early apoptosis, B2: late apoptosis); (C) HUVECs were treated with different concentration of glucose and GLP-1 (C1: early apoptosis, C2: late apoptosis) as compared with the control group (^∗^*P* < 0.05, compare with C group; ^#^*P* < 0.05, compare with H group). Note: Ctrl: control (5.5 mmol/L glucose); H: high glucose (33 mmol/L glucose).

Our results showed high glucose could promote apoptosis as shown in Fig. 3. Compared with the control group, both early and late phase of apoptosis of HUVECs with high glucose treatment (33 and 47 mmol/L) were significantly increased (Fig. 3A). Compared with the control group, the level of apoptosis was decreased in the group that was treated with GLP-1. Early apoptosis was significantly decreased in the groups that were treated with various concentrations of GLP-1 (0.01, 0.1, 1, and 10 nmol/L). Late apoptosis was significantly decreased in the cells treated with GLP-1 (1 and 10 nmol/L) (Fig. 3B). The GLP-1 treatment also reduced the level of apoptosis in high glucose group, but it was not completely consistent between early and late stage (Fig. 3C).

### Protective Effects of GLP-1 to Oxidative Stress

**FIGURE 4:**
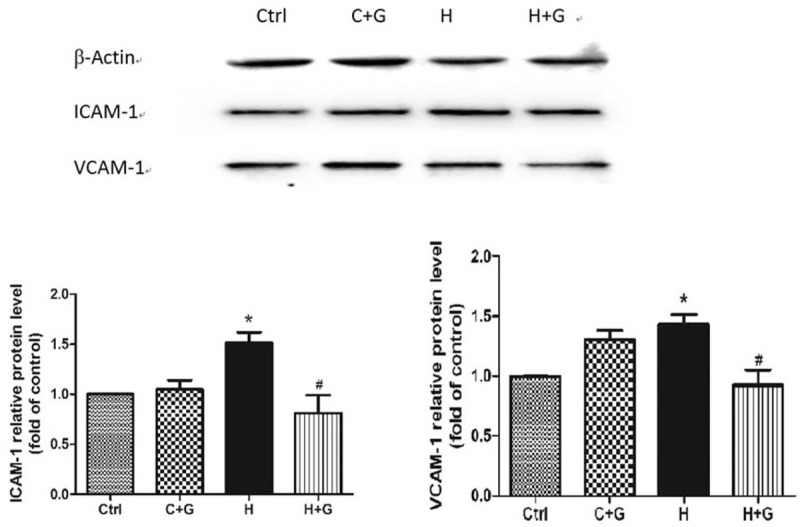
The GLP-1 decrease of ICAM-1 and VCAM-1 that was induced by high glucose in HUVECs as compared with the control group (^∗^*P* < 0.05, compare with C group; ^#^*P* < 0.05, compare with H group). Ctrl: control (5.5 mmol/L glucose); C + G: control + 10 nmol/L GLP-1; H: 33 mmol/L glucose; H + G: 33 mmol/L glucose + 10 nmol/L GLP-1.

We further investigated whether GLP-1 actually improved the oxidative stress in HUVECs. We studied the ICAM-1 and VCAM-1 protein expression in HUVECs. GLP-1 inhibited ICAM-1 and VCAM-1 protein expression in HUVECs. Compared with the control group, the expression of ICAM-1/VCAM-1 was increased in high glucose groups and then significantly declined with GLP-1 treatment (Fig. 4).

### GLP-1 Inhibited Oxidative Stress-Induced Apoptosis Probably Through JNK Signaling Pathway in HUVECs

**FIGURE 5:**
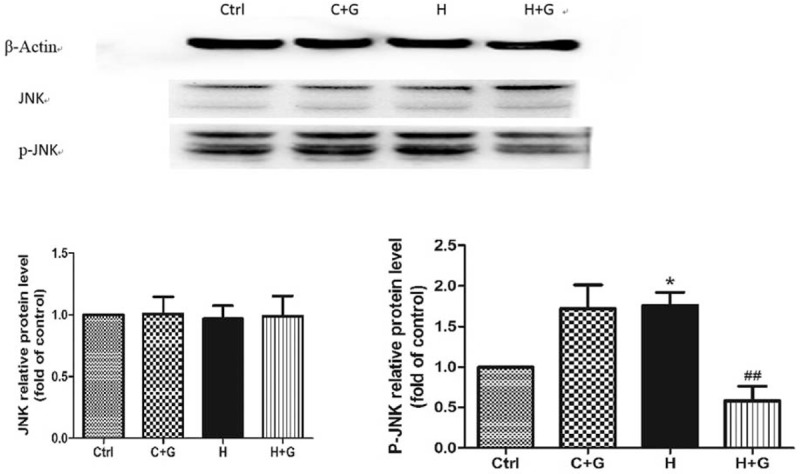
The GLP-1 decrease in p-JNK that was induced by high glucose in HUVECs as compared with the control group (^∗^*P* < 0.05). Ctrl: control (5.5 mmol/L glucose); C + G: control + 10 nmol/L GLP-1; H: 33 mmol/L glucose; H + G: 33 mmol/L glucose + 10 nmol/L GLP-1.

**FIGURE 6:**
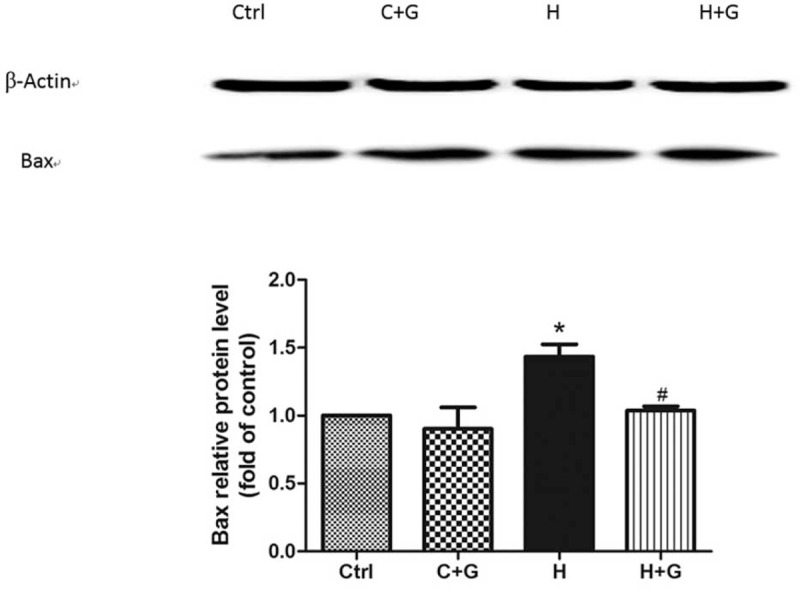
The GLP-1 decrease in Bax that was induced by high glucose in HUVECs as compared with the control group (^∗^*P* < 0.05, compare with C group; ^#^*P* < 0.05, compare with H group). Ctrl: control (5.5 mmol/L glucose); C + G: control + 10 nmol/L GLP-1; H: 33 mmol/L glucose; H + G: 33 mmol/L glucose + 10 nmol/L GLP-1.

It has been reported that high glucose could easily increase the proapoptotic effects on HUVECs, which might account for the increased oxidative stress and the activation of JNK signal transduction pathway.^19^ We therefore investigated the involvement of this intracellular cascade in the GLP-1 protective effect. The p-JNK was significantly increased in the control group that was treated by GLP-1 (Fig. 5). Compared with the control group, the p-JNK was significantly decreased in the group that was treated with high glucose followed by GLP-1 intervention. However, the expression of JNK was not significantly different as compared with the high glucose groups (Fig. 5). Meanwhile, the expression of Bax was significantly decreased in the group that was treated with high glucose after GLP-1 intervention (*P* = 0.046) (Fig. 6).

## DISCUSSION

In the present study, we provided evidence that GLP-1 protected the endothelial cells from high glucose-induced oxidative damage and apoptosis. GLP-1 is a novel therapeutic agent for the treatment of diabetes mellitus. It was reported that GLP-1 could improve cardiovascular damage^10,20,21^ and endothelial function.^22^

It was reported that glucose-induced mitochondria production of ROS had some correlation with hyperglycemia-mediated complications of diabetes.^23^ The mitochondria is known to play a key role in activating the apoptotic ROS, produced from the mitochondrial transport chain. ROS is involved in apoptosis induced by chronic exposure to high glucose in many cell types.^19,24,25^ Our observations of glucose-induced apoptosis in HUVECs were consistent with these in vitro studies.

Our model system with primary HUVECs supported the hypothesis that GLP-1 directly improved the endothelial oxidative injury and reduced the level of apoptosis. Our data suggested that compared with high glucose group, GLP-1 treatment significantly reduced the ROS level in the groups that were treated with high glucose. In advanced glycation end products (AGE)-exposed HUVECs, GLP-1 decreased the generation of ROS and subsequently reduced vascular cell adhesion molecule-1 mRNA levels.^26^ In this study, GLP-1 inhibited VCAM-1 that was induced by high glucose in HUVECs. The expression of VCAM-1 was significantly declined. Therefore, we concluded that GLP-1 improved the endothelial cell oxidative damage through a reduction in the production of ROS.

GLP-1 has been shown to improve the endothelial vasodilatation dysfunction in type II diabetic patients with coronary heart disease.^22^ GLP-1 administration not only attenuated endothelial cell dysfunction in diabetic patients but also inhibited the induction of tumor necrosis factor (TNF-α)-mediated plasminogen activator inhibitor type-1 (PAI-1) in human vascular endothelial cells.^27^ The GLP-1 analog, Liraglutide, just like GLP-1 exerted an antiinflammatory effect on vascular endothelial cells by increasing nitric oxide production and suppressing NF-κB activation.^28^ Our result also showed that GLP-1 treatment had a protective effect on production of ROS and oxidative injury induced by high glucose.

GLP-1 has been shown to reduce apoptosis in many types of cells.^29–31^ It has been reported that the main effects of GLP-1 mediated the antiapoptotic action through a cAMP- and PI3K-dependent signaling pathway.^32^ In pancreatic β-cells, GLP-1 inhibited apoptosis and stimulated the survival and proliferation. Furthermore, the effect of GLP-1 was suppressed by inhibitors of EGFR (AG1478) and PI3-kinase (LY294002).^33^ However, GLP-1 could protect coronary artery endothelial cells from lipoapoptosis by PKA, PI3K, eNOS, p38 MAPK, and JNK pathways^34^ and it was reported that PI3K/Akt signaling was not activated by GLP-1 in other endothelial cells and the inhibition of this pathway could not abolish the GLP-1 protective effects.^35^ Thus, we concluded that GLP-1 manifested its protective effects through different pathways in different cell types.

This study was undertaken to investigate the biological activity and the mechanism of action of GLP-1 in the HUVEC injury induced by high concentration of glucose. The results showed that the level of apoptosis in control group treated by GLP-1 was decreased, the early apoptosis was significantly decreased in groups that were treated with various concentrations of GLP-1 and late apoptosis was significantly decreased in the cells treated with GLP-1 (1 and 10 nmol/L). GLP-1 treatment also reduced the level of apoptosis in the high glucose group. This process may be mediated by a decreased expression of the p-JNK and Bax. At the same time, the result of MTT assay showed that GLP-1 increased the cell viability in a dose-dependent manner in different GLP-1 treated groups.

In this study, we investigated the cellular effect of high glucose in HUVECs and the possible function of GLP1 in different concentrations of glucose treated cells. However, the relative upstream signaling pathway molecules of JNK-dependent in HUVECs could not be studied. This will be the next step of our work.

In conclusion, we showed that GLP-1 improved the endothelial cell oxidative damage and apoptosis induced by ROS via high glucose mediated. p-JNK and Bax apoptotic proteins were also regulated by GLP-1. These findings could provide further evidence that GLP-1 had the effects on endothelial cell damage induced by high glucose and this could be a starting point to investigate the protective mechanisms to improve the diabetes.
